# Effectiveness of a scalable, remotely delivered stepped-care intervention to reduce symptoms of psychological distress among Polish migrant workers in the Netherlands: study protocol for the RESPOND randomised controlled trial

**DOI:** 10.1186/s12888-023-05288-5

**Published:** 2023-11-02

**Authors:** Rinske Roos, Anke B. Witteveen, José Luis Ayuso-Mateos, Corrado Barbui, Richard A. Bryant, Mireia Felez-Nobrega, Natasha Figueiredo, Raffael Kalisch, Josep Maria Haro, David McDaid, Roberto Mediavilla, Maria Melchior, Pablo Nicaise, A-La Park, Papoula Petri-Romão, Marianna Purgato, Annemieke van Straten, Federico Tedeschi, James Underhill, Marit Sijbrandij, the RESPOND Consortium

**Affiliations:** 1 Department of Clinical, Neuro- and Developmental Psychology and WHO Collaborating Center for Research and Dissemination of Psychological Interventions, VU University, Amsterdam, Netherlands; 2 Department of Psychiatry, Universidad Autónoma de Madrid (UAM), Madrid, Spain; 3 Department of Psychiatry, La Princesa University Hospital, Instituto de Investigación Sanitaria Princesa (IIS-Princesa), Madrid, Spain; 4 Centro de Investigación Biomédica en Red de Salud Mental (CIBERSAM), Instituto de Salud Carlos III (ISCIII), Madrid, Spain; 5 Department of Neuroscience, Biomedicine and Movement Sciences, Section of Psychiatry, WHO Collaborating Centre for Research and Training in Mental Health and Service Evaluation, University of Verona, Verona, Italy; 6 School of Psychology, University of New South Wales, Sydney, NSW Australia; 7 Research and Development Unit, Parc Sanitari Sant Joan de Déu, Barcelona, Spain; 8 Equipe de Recherche en Epidémiologie Sociale (ERES), Institut Pierre Louis d’Epidémiologie et de Santé Publique (IPLESP), INSERM, Sorbonne Université, Faculté de Médecine St Antoine, Paris, France; 9 Leibniz Institute for Resilience Research (LIR), Mainz, Germany; 10 Focus Program Translational Neuroscience (FTN), Neuroimaging Center (NIC), Johannes Gutenberg University Medical Center, Mainz, Germany; 11 Care Policy and Evaluation Centre, Department of Health Policy, London School of Economics and Political Science, London, UK; 12 Institute of Health and Society (IRSS), Université Catholique de Louvain, Brussels, Belgium; 13 Independent Research Consultant, Brighton, UK

**Keywords:** International migrant workers, Randomized controlled trial, Scalable interventions, Non-specialist healthcare workers, Stepped-care, Common mental health disorders, Digital biomarkers, Hair steroid hormone concentrations

## Abstract

**Background:**

The COVID-19 pandemic has negatively affected the mental health of international migrant workers (IMWs). IMWs experience multiple barriers to accessing mental health care. Two scalable interventions developed by the World Health Organization (WHO) were adapted to address some of these barriers: Doing What Matters in times of stress (DWM), a guided self-help web application, and Problem Management Plus (PM +), a brief facilitator-led program to enhance coping skills. This study examines whether DWM and PM + remotely delivered as a stepped-care programme (DWM/PM +) is effective and cost-effective in reducing psychological distress, among Polish migrant workers with psychological distress living in the Netherlands.

**Methods:**

The stepped-care DWM/PM + intervention will be tested in a two-arm, parallel-group, randomized controlled trial (RCT) among adult Polish migrant workers with self-reported psychological distress (Kessler Psychological Distress Scale; K10 > 15.9). Participants (*n* = 212) will be randomized into either the intervention group that receives DWM/PM + with psychological first aid (PFA) and care-as-usual (enhanced care-as-usual or eCAU), or into the control group that receives PFA and eCAU-only (1:1 allocation ratio). Baseline, 1-week post-DWM (week 7), 1-week post-PM + (week 13), and follow-up (week 21) self-reported assessments will be conducted. The primary outcome is psychological distress, assessed with the Patient Health Questionnaire Anxiety and Depression Scale (PHQ-ADS). Secondary outcomes are self-reported symptoms of depression, anxiety, posttraumatic stress disorder (PTSD), resilience, quality of life, and cost-effectiveness. In a process evaluation, stakeholders’ views on barriers and facilitators to the implementation of DWM/PM + will be evaluated.

**Discussion:**

To our knowledge, this is one of the first RCTs that combines two scalable, psychosocial WHO interventions into a stepped-care programme for migrant populations. If proven to be effective, this may bridge the mental health treatment gap IMWs experience.

**Trial registration:**

Dutch trial register NL9630, 20/07/2021, https://www.onderzoekmetmensen.nl/en/trial/27052

**Supplementary Information:**

The online version contains supplementary material available at 10.1186/s12888-023-05288-5.

## Introduction

Large-scale epidemics of infectious diseases have been associated with a substantial burden on the mental health of the population [1]. Although recent studies have not found convincing evidence for substantial increases in mental health symptoms during the COVID-19 pandemic compared to the pre-pandemic period [2], trajectory studies show that the mental health of vulnerable groups such as women, young people, people with pre-existing physical ill-health, or those experiencing socioeconomic difficulties deteriorated more than the general population during the COVID-19 pandemic [3, 4].

International migrant workers (IMWs) can be viewed as another group vulnerable to the consequences of the COVID-19 pandemic. IMWs are migrants of working age, who at some point were part of the labour force of the country they migrated to [5]. Globally, there are 169 million IMWs, of which two-thirds reside in high-income countries [5]. In the Netherlands, there are about one million IMWs, with over half of them coming from the European Union, primarily from Poland (CBS, 2022). IMWs often have a vulnerable position in society, working in so-called 3-D jobs: dirty, dangerous, and demanding (or demeaning or degrading) jobs [6]. Compared to non-migrant workers, IMWs are more likely to work in essential, low-skilled occupations, have temporary contracts, work longer hours for lower wages, are willing to take on greater risks and have jobs that are not suitable for remote working [6, 7]. Most often, they work in the service sector (e.g. wholesale and retail, transportation and storage), followed by industry and agriculture [5]. During the pandemic, additional challenges for IMWs were, amongst others, limited social protection, high risk of exposure to and transmission of COVID-19, and impending job loss, in turn, leading to economic hardship and loss of housing (often provided by the employer) [8].

Prior to the COVID-19 pandemic, mental health problems were already one of the most commonly reported work-related health problems among IMWs [9, 10]. IMWs have been found to develop more symptoms of anxiety and depression than non-migrant workers [11]. These common mental health problems have been exacerbated by the ongoing COVID-19 pandemic [12]. Despite this mental health burden, access to specialist health care is limited [10, 13]. IMWs can face various barriers related to seeking mental health services (e.g. lack of awareness of services, stigma) and to accessing the existing services (e.g. language differences, lack of culturally appropriate services) [14, 15]. In light of the COVID-19 pandemic, there is thus an even higher need for psychosocial interventions for IMWs targeting the most notable symptoms of psychological distress, such as anxiety, depression, and posttraumatic stress disorder (PTSD) [16, 17].

Scalable strategies and interventions such as those developed by the World Health Organization (WHO) may bridge the mental health treatment gap in vulnerable populations such as IMWs. These interventions are scalable because they are simplified and short versions of evidence-based psychological interventions for common mental disorders and can be delivered as (guided) self-help interventions (e.g. a book or online format) and/or by trained and supervised non-specialist mental health care workers [18, 19]. Since the onset of the COVID-19 pandemic, there has been growing shift to the remote delivery of mental health services due to physical distancing and lockdowns [4]. Human-guided digital interventions seem to be equally effective to face-to-face psychotherapy for the treatment of common mental health symptoms such as anxiety and depression [20]. Policy makers, mental health professionals and service users have expressed interest in continuing with this remote delivery in the absence of pandemic-related measures [21].

For this study, two WHO scalable interventions have been combined to be delivered remotely as a stepped-care intervention. In stepped-care interventions, individuals first receive an evidence-based, low-intensity treatment, i.e. a treatment requiring less of the individual’s and the professional’s time and which is less expensive [22]. As patients’ progress is monitored, those not (significantly) responding to treatment step up to a treatment of higher intensity [22]. In this way, stepped-care interventions have the potential to reach more people at the cost of fewer resources. Often, (guided) self-help treatments are used as a first step in stepped-care interventions, showing comparable effectiveness to face-to-face interventions [23].

In this RCT of IMWs in the Netherlands, participants in the intervention group are offered a two-step, stepped-care intervention. The first step is a digitalized guided self-help web application (web app) of Doing What Matters in times of stress (DWM), an illustrated self-help book that is part of Self-Help Plus (SH +) [24]. SH + is a guided self-help intervention based on acceptance and commitment therapy (ACT) that is delivered in five 2-h sessions to groups of 20–30 people. So far, SH + has been evaluated among refugee populations, showing overall beneficial effects in improving self-identified problems and well-being [25]. For this project, DWM has been adapted for delivery in a digital smartphone-based format [26]. The second step is Problem Management Plus (PM +), a transdiagnostic psychological intervention based on cognitive behavioural therapy (CBT) that addresses common mental health problems (e.g. depression, anxiety, stress) and self-identified practical problems (e.g. unemployment). Over five weekly face-to-face remotely delivered videoconferencing sessions, PM + teaches strategies to manage psychosocial problems [27]. In previous randomized controlled trials (RCTs), PM + has been found to be effective in reducing psychological distress in low-income settings [28, 29] and Syrian refugees in the Netherlands [30, 31]. In addition to DWM and PM + , all participants, i.e. participants in both the intervention and control group, receive psychological first aid (PFA). PFA consists of humane, supportive, and practical help for individuals who have experienced a traumatic event [32]. This stepped-care programme has also been found to be effective among healthcare workers experiencing psychological distress in the initial pandemic hotspots [33].

Recent advances have been made in examining both digital markers and biomarkers either as correlates or as secondary treatment outcomes. Traditional assessment of psychological well-being and distress in IMWs through (online) questionnaires can be burdensome and time-consuming, while recently developed non-invasive, low-burden digital phenotyping measures that integrate voice, speech, movement and facial expression data from smart devices (e.g. smartphones) may be a promising and scalable way for assessing psychological wellbeing (e.g. depression, PTSD) [34–36]. Notably, altered speech and vocality, reduced facial expressivity and movement have been found in major depressive disorder and psychopharmacological treatment has demonstrated restored levels of digital markers (e.g. increased head movement) and a decrease in anger and fear facial expressions [37, 38]. Similarly, neuroendocrine correlates such as cortisol can be used as a non-invasive biomarker of physiological responses to chronic stressors. Hair cortisol concentrations (HCC) reflect hormone release over longer time intervals, as they indicate hormone secretion over several months [39]. Studies have found that HCC is related to PTSD, depression and anxiety disorders [40, 41] or with perceived job insecurity and work stress [42, 43]. In recent years, HCC has also been used as a secondary outcome of psychological interventions [44, 45].

This paper presents the study protocol for a randomised controlled trial in the Netherlands to examine the (cost-)effectiveness of the remotely delivered stepped-care DWM/PM + programme adapted for distressed IMWs living in the Netherlands. The final stage will consist of a process evaluation to assess the feasibility and acceptability of the intervention.

## Methods

### Study aim and design

**Fig. 1:**
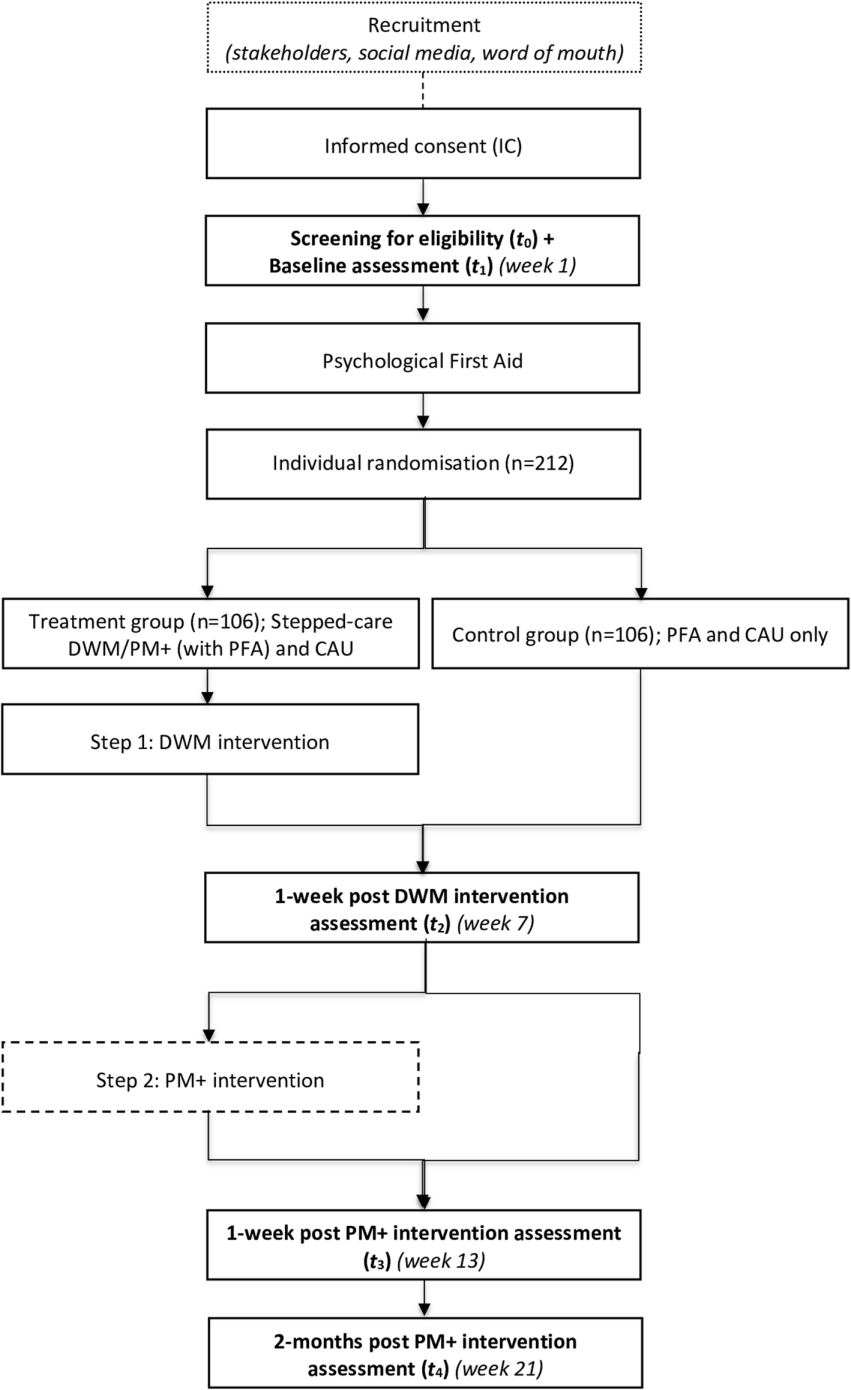
Flowchart of the randomized controlled trial

This study is part of the EU Horizon2020 RESPOND project, which aims to improve the preparedness of European mental health care systems in the face of future pandemics. The primary objective of the current study is to evaluate the (cost-)effectiveness, feasibility, and acceptability of the culturally and contextually adapted DWM/PM + stepped-care program among Polish migrant workers living in the Netherlands throughout the COVID-19 pandemic in terms of mental health outcomes, resilience, wellbeing, and costs to health systems and society. This will be done by conducting a single-blind, two-arm, parallel-group, superiority RCT with a 1:1 allocation ratio in which a remotely delivered stepped-care programme with PFA and care-as-usual (enhanced care-as-usual or eCAU) will be compared to PFA with CAU (eCAU) only. The primary endpoint is a composite measure of depression and anxiety at 21 weeks from baseline assessment (*t*_4_). Figure 1 shows a flowchart of the study design. In addition, we will explore barriers and facilitators to treatment engagement and adherence and opportunities for scaling up among IMWs in the Netherlands, as well as the implementation outcomes of the stepped-care programme. This will be done by a mixed-methods process evaluation following the RCT.

### Study setting

This study takes place in the Netherlands and is conducted by VU University Amsterdam in collaboration with a local mental health care organisation specialized in the delivery of mental health care to Polish people [GGZ Keizersgracht]. The DWM/PM + programme is delivered fully remotely in the participants’ language (i.e. Polish); both participants and those delivering the intervention join the programme from their own (private or work) environments.

### Participants

#### Inclusion criteria

- age 18 years or older;
- currently living in the Netherlands (no duration of stay required; they may have arrived after most pandemic restrictions had been lifted);
- having psychological distress, as indicated by the Kessler Psychological Distress Scale (K10) with a score of > 15.9 (see screening measure);
- having sufficient mastery (written and spoken) of the language in which the programme is delivered (i.e. Polish);
- having access to an electronic device with Internet access to follow the programme.

#### Exclusion criteria

- planning to permanently move abroad within the next 6 months, i.e. before the last quantitative assessment;
- having acute medical conditions (requiring hospitalization);
- imminent suicide risk, or expressed acute needs or protection risks requiring immediate follow-up;
- having a severe mental disorder (e.g. psychotic disorder, substance dependence);
- having a severe cognitive impairment (e.g. severe intellectual disability, dementia);
- receiving specialist psychological treatment (e.g. eye movement desensitization and reprocessing, CBT);
- using psychotropic medication for less than 2 months or change in dosage in the past 2 months.

### Procedure

#### Recruitment

Participants will be recruited in various ways, e.g. through NGOs, general practitioners, municipalities, Polish churches, Polish supermarkets, and social media (e.g. Facebook, Instagram). To avoid potential coercion, people interested in participating in the project are invited to reach out to the research team themselves (i.e. by e-mail, telephone, or through social media). Once they reached out, the research team contacts all potential participants by phone. If they are interested, both a digital and paper informed consent form (ICF) will be sent. Permission to record DWM phone calls and/or PM + sessions for fidelity checks and digital marker analysis, and/or to provide a hair sample (see other measures), are all optional.

Once the participant has given informed consent, an online screening session is scheduled. All participants that indicate on the ICF that they are willing to give a hair sample will receive a hair sample kit consisting of instructions on how to take the hair sample, a (paper) hair questionnaire, and a prepaid envelope to return it.

#### Screening

Screening (*t*_0_) will be conducted through a video call (MS Teams, 20–30 min) with a Polish speaking research assistant. Screening consists of an online self-report questionnaire (sociodemographic questions and K10) followed by exclusion questions that are asked directly to the participant (e.g., “did you receive any mental health diagnosis?”) or answered by the research assistant (e.g., “does the participant follow the conversation?”). Screening is ended as soon as a participant screens out based on any inclusion or exclusion criterion. Participants who indicated on the ICF that they were willing to give a hair sample but score too low on the K10 are still asked to give a hair sample at baseline and to complete the baseline assessment (*t*_1_).

#### Assessments

After screening, data is collected through self-report assessments at four time points: *t*_1_ (baseline, week 1 – sent at the end of screening), *t*_2_ (post-DWM, week 7), *t*_3_ (post-PM + , week 13), and *t*_4_ (follow-up, week 21) with the online programme Castor Electronic Data Capture (EDC) [46]. Each digital assessment with questionnaires will be sent to participants via e-mail and can be completed through any device with Internet access (e.g. smartphone, laptop, tablet). Total completion time of each digital assessment is about 30 min (see ‘Study measures’).

Participants have 14 days to complete the questionnaire When an assessment remains incomplete, participants will receive a maximum of 3 reminders on days 2, 5, and 10 through different communication channels to promote participant retention. Hair samples and (paper) questionnaires are collected at *t*_1_ and *t*_4_ through regular mail. Once the research team receives the hair sample and the hair questionnaire, answers are entered in Castor EDC as well. Participants receive a 10-euro gift voucher for each completed assessment with a maximum of 4 vouchers (*t*_1_-*t*_4_), regardless of whether they send a hair sample.

#### Assessors

The consent and screening procedure will be carried out by trained research assistants who are fluent in Polish. Research assistants will be blinded to participant allocation to the intervention or control group, by restricting their user rights in Castor EDC. This way, they can assist participants during the assessments if needed. Blinding may be revealed in case of suicidal ideation (see screening measures).

#### Randomization

Participants (*N *= 212) will be randomized into the PFA/DWM/PM + CAU (intervention) (*n *= 106) or PFA/CAU only (control) (*n* = 106) group using a four and six block design via Castor EDC.

#### Trial status

This RCT started enrolling on 03 June 2022. Currently, 138 participants have been enrolled.

#### Process evaluation

After the RCT, participants who gave permission for follow-up research will be approached to participate in the process evaluation. A qualitative process evaluation will be conducted to assess the satisfaction and acceptability of the DWM/PM + programme and to identify barriers and facilitators to treatment engagement and adherence. Additionally, this evaluation aims to explore opportunities for scaling up the implementation of the DWM/PM + programme for IMWs within the existing healthcare system. This evaluation will be conducted through semi-structured interviews with key informants, including DWM/PM + participants (*n* = 20), family members/close persons of participants (*n* = 12), and stakeholders (e.g. (mental) health practitioners, policy makers). DWM/PM + participants will consist of participants who completed DWM and improved, who completed PM + and improved, who completed PM + and did not improve, who dropped out during DWM, and who dropped-out during PM + . Additionally, a focus group discussion (FGD) will be conducted with helpers and trainers/supervisors (*n* = 6–8) (see *‘*Stepped-care programme*’*). Participants from the RCT and their family members/close friends will each receive a 20 euro gift voucher per interview.

### Study measures

Table 1 provides an overview of all study measures. Participants need to complete the items of the K10, the primary outcome (PHQ-ADS), and the suicide screening, in order to continue the assessment. The rest of the questionnaires are non-mandartory and participants can skip items and sections.

**Table 1:**
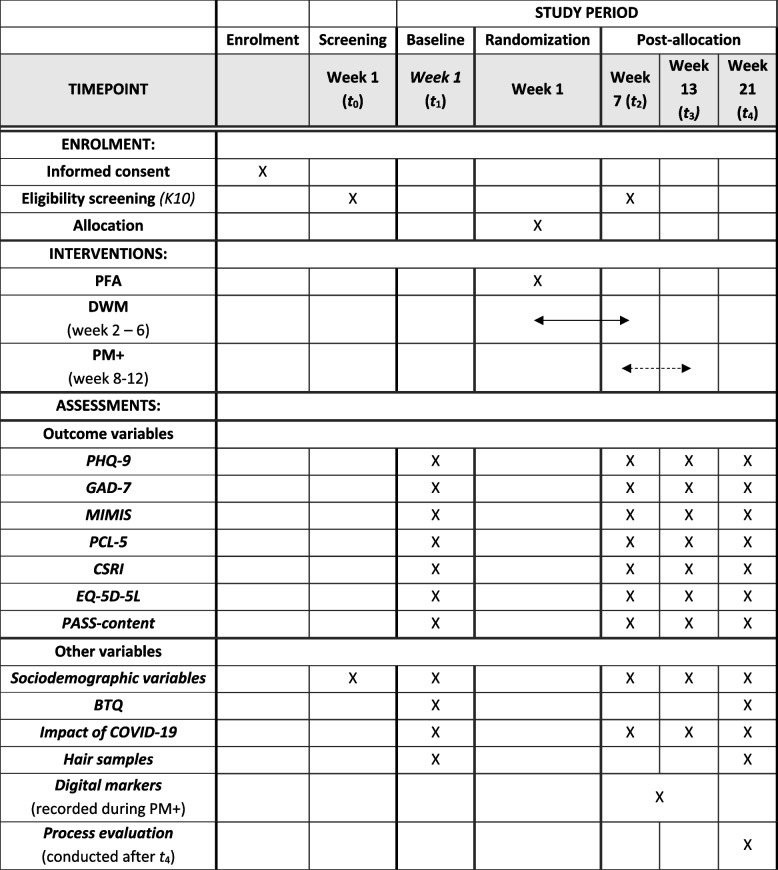
Schedule of enrolment, interventions and assessments

#### Screening measures

Psychological distress will be measured with the K10 [47]. The K10 consists of ten items that focus on anxiety and depression-related distress over the past 30 days. Items are scored on a five-point Likert scale (1–5), giving a total score of 10–50 with higher scores representing higher levels of distress. In line with previous research [48, 49] a cut-off score of > 15.9 is used, which indicates psychological distress [50]. The K10 is validated in various languages (e.g. Dutch) [51].

 Suicidal ideation will be assessed with the suicidal thoughts interview from the PM + manual (*t*_0_*,* screening) [52] or with a screening question used in a previous trial testing an e-health intervention (*t*_2_-*t*_4_) [53]. If a participant answers positively to this screening question (*t*_2_-*t*_4_), a message will appear with information on where to get help and stating that a member of the research team will reach out to the participant. Once a phone call has been scheduled with the participant, an assessor will administer the suicidality module of the Mini-International Neuropsychiatric Interview (MINI) [54]. Suicidal ideation is not assessed during *t*_1_ as this directly follows screening. Cognitive impairment will be assessed with the observation checklist from the PM + manual.

#### Primary outcome measure

The primary outcome and endpoint is a combined measure of depression and anxiety at week 21 (*t*_4_, 2-month follow-up) as indicated by the Patient Health Questionnaire Anxiety and Depression Scale (PHQ-ADS). The PHQ-ADS is a validated measure of the combined sum score of the Patient Health Questionnaire depression module (PHQ-9) [55] and Generalized Anxiety Disorder (GAD-7) [56] questionnaire (see secondary outcomes). Answer options for both the PHQ-9 and GAD-7 are ‘not at all’ (0), ‘several days’ (1), ‘more than half the days’ (2), and ‘nearly every day’ (3) with a sum score of 0–48.

#### Secondary outcome measures

Symptoms of depression and anxiety during the preceding two weeks will be measured by the nine-item PHQ-9 [55] and the seven-item GAD-7 [56] respectively. Answers on both questionnaires are scored on a 0–3 Likert scale (PHQ-9: range 0–27; GAD-7: range 0–21) with higher scores indicating higher symptomatology. For both questionnaires, a cut-off score of 10 will be used [56, 57]. In addition to the nine items, the PHQ-9 asks: “If you checked off any problems, how difficult have these problems made it for you to do your work, take care of things at home, or get along with other people?” which is answered on a four-point Likert scale ranging from ‘not difficult at all’ to ‘extremely difficult’. The Polish version of the PHQ-9 has been validated [58]. The Polish version of the GAD-7 has not been validated yet but has recently been used in various studies to assess anxiety during the COVID-19 pandemic in Poland and shown high internal consistency [59–61].

Symptoms of PTSD during the past month will be measured by the 8-item PCL-5 [62], which is a shortened version of the 20-item PTSD Checklist for DSM-5 (PCL-C) [63]. Items are rated on a 0–4 scale (0–32), with higher scores indicating higher levels of symptoms. The Polish version of the 20-item PCL-5 has been found to have good psychometric properties [64].

Outcome-based resilience will be operationalised as an individual’s deviation from the sample-normalised stressor reactivity (SR) line. To this end, a measure of stressor exposure has been developed based on the Mainz Inventory of Microstressors (MIMIS) [65] and similar measures [66]. The stressor lists were further adapted to fit the target population and shortened to minimize burden of participants. The questionnaire consists of 22 items measuring life events (three items), and general (six items), COVID-related (five items) and migrant specific (eight items) stressors. Life events are measured on a five-point Likert scale (0–4) rating severity and stressors are measured on a four-point Likert scale (0–3) rating how often something occurred. The sample’s normative stressor reactivity will be calculated by regressing stressor exposure (E; as measured by the stressor lists) against mental health symptoms (P; as measured by PHQ-ADS, PHQ-9, GAD-7). For the purpose of building the SR score, the E score that explains the most variance of mental health symptoms will be used. The best fitting regression model will be taken as the sample’s E-P line. Individual SR scores are then calculated as the residuals to the E-P line, where a positive deviation denotes less resilient outcomes and negative scores more resilient outcomes, respectively.

Positive appraisal style, a factor leading to resilience, will be measured with the Positive Appraisal Style Scale, content-focused (PASS-content) [67]. It consists of 12 items focusing on how participants usually act, feel, and think in stressful situations. Scores range from 1 (never) to 4 (almost always; total range 12–48).

Quality of life will be measured by the EuroQol 5-dimensional descriptive system—5-level version (EQ-5D-5L) which consists of the EQ-5D (part 1) and EQ-VAS (part 2) [68]. The EQ-5D consists of five items rating the level of impairment across the dimensions of mobility, self-care, usual activities, pain/discomfort, and anxiety/depression. Each dimension has 5 levels: no problems, slight problems, moderate problems, severe problems and extreme problems. The EQ-VAS consists of a visual, analogue scale with as endpoints ‘the best’ [1] and ‘the worst’ [5] health you can imagine. The EQ-5D-5L has been used widely and is available in over 150 languages, including Polish, also for collection by laptop, tablet or Castor EDC [69].

To examine cost-effectiveness, health service utilization and the effect of ill-health on participants’ and their family/friends’ employment over the past 2 (*t*_2_, *t*_3_, *t*_4_) or 3 (*t*_1_) months will be measured with an adapted version of the Client Service Receipt Inventory (CSRI) [70]. The CSRI has been further adapted to fit the target population of this study. Health service utilization focuses on 12 types of health care providers, as well as in- and out-patient hospital services. If utilization of a health service is indicated, follow-up questions will be asked regarding the contact, e.g. number of visits, type (in-person/online) and duration of contact, location (Netherlands/Poland), travel and waiting time, and travel costs. Additionally, trial costs (e.g. training, supervision, intervention facilitator time) are collected.

#### Other measures

Sociodemographic information is collected at all timepoints. During the screening, basic information (e.g. gender, age, education), information regarding migration (e.g. time spent in the Netherlands), and work (e.g. sector, working hours, income source, contract type) are collected. At baseline, further information is collected (e.g. relationship status, living situation). Potentially fluctuating sociodemographic information is recollected at follow-up assessments.

Exposure to potentially traumatic events will be assessed using a shortened version of the Brief Trauma Questionnaire (BTQ) [71]. The BTQ comprises 10 items, each corresponding to an event that can be indicated as either having occurred or not. To accommodate follow-up, minor adaptations were made to the BTQ.

Impacts of COVID-19 will be assessed with a questionnaire based on other COVID-19 questionnaires [72]. It consists of 16 items focusing on various aspects of the COVID-19 pandemic (e.g. COVID-19 infection, adherence to and consequences of COVID-19 regulations) and an additional item on vaccination status at baseline assessment.

After the trial, various implementation indicators, e.g. reach, dose, resource use, and costs (training, staff, recruitment etc.), will be assessed. Furthermore, based on the implementation indicators and fidelity data the incremental costs per change in the primary outcome and quality of life will be estimated.

Hair cortisol concentrations (HCC), cortisone, dehydroepiandrosterone (sulphate) (DHEA(S)), testosterone, and progesterone levels will be analysed through hair samples. In about 100 hair strands, cut as close as possible from the scalp at the vertex posterior, the 3cm hair from the scalp will be analysed for cumulative HCC, cortisone, and DHEA(S) levels [39]. Based on an average hair growth rate of 1 cm/month [73], this will give an indication of physiological stress levels over 3 months. Participants will be instructed to obtain a hair sample with the assistance of another person. As support, an instruction video has been created. In addition, participants are asked to complete a hair questionnaire to assess several relevant hair-specific characteristics (e.g. on hair washing, hair treatments and on substance or medication use such as corticosteroids).

Video records of PM + sessions will be analysed for digital markers of facial activity (e.g. happiness, sadness, and fear), voice (e.g. speech prevalence), and movement (e.g. head pitch). This will be done using open-source tools based on the analysis by Galatzer-Levy et al. [74].

#### Translation and adaptation of the study measures

When available, instruments translated and/or validated in Polish were selected. In line with WHO guidelines on the translation and adaptation of research instruments [75], instruments without existing Polish translations were translated and back-translated. In case of discrepancies in translation, items were discussed by the translators to reach consensus.

## Interventions

### Psychological First Aid (PFA)

Two to five days after screening, all participants will receive Psychological First Aid (PFA) by telephone. PFA is a support strategy for someone who is experiencing psychological distress and may need support. It consists of various themes, such as assessing and addressing (basic) needs and concerns, providing practical care and support, listening and comforting people, connecting them to information and protecting them from further harm [32]. At the end of the phone call, participants will be informed of the group they have been randomized into.

### Stepped-care programme

Participants in the intervention group are offered a stepped-care programme. All participants are offered step 1. Participants who still report elevated levels of psychological distress after step 1 (i.e. score > 15.9 on the K10 at *t*_2_) are also offered step 2.

#### Step 1: Doing What Matters in times of stress (DWM)

The DWM web app consists of five modules with accompanying audio exercises corresponding to each of the five chapters of the SH + self-help book: (1) grounding, (2) unhooking, (3) acting on your values, (4) being kind, and (5) making room. Each week, a new module unlocks. Based on Step-by-Step, a guided e-mental health intervention [53], participants receive a total of six weekly 15-min support calls from a non-specialist (lay) helper *(see ‘*Helpers*’)*: one welcome call, and a total of five follow-up calls – one at the end of each module. During these calls, the previous module(s) and exercise(s) are discussed and participants have the opportunity to ask questions. Helpers have also limited access to participants’ metadata, such as if a participant has logged into the web app and when they finished a module. Additionally, the web app has a chat support system through which participants can chat with their helper at the weekly support moment in case they are unable to have a phone call.

#### *Step 2: Problem Management Plus (PM* +*)*

PM + teaches four strategies: (1) stress management, (2) problem solving, (3) behavioural activation, and (4) strengthening social support, and has a psycho-education component [27]. PM + is delivered over five weekly sessions with a non-specialist (lay) helper *(see ‘*Helpers*’)*. For this project, PM + has been adapted to be delivered in individual 60 (instead of 90) minute video-call sessions (MS Teams). Before implementation of the stepped-care interventions, a qualitative sub-study is performed to assess the main daily-life problems and psychosocial care needs of IMWs in the Netherlands, to contextually adapt both interventions [76]. Data collected for this qualitative assessment is conducted following Module 1 of the Design, Implementation, Monitoring and Evaluation (DIME) research model, which consists of free listing interviews, key-informant interviews with both IMWs and professionals and a focus group discussion [77]. Core elements of the interventions remained the same, while case examples and pictures were adapted. Details of this adaptation process will be published elsewhere.

#### Helpers

Helpers are Polish migrants living in the Netherlands. Participants are linked to a helper who delivers all calls in DWM or all sessions in PM + . For participants who continue to PM + , this may be the same helper as they had for DWM, but this is not necessary. Helpers delivering the DWM support calls and PM + sessions will be Polish people living in the Netherlands. They have been trained in the intervention by mental health professionals, who themselves have been trained by so-called Master trainers in both the intervention and the supervision process.

Training of trainers (ToT) was online and consisted of a two-day DWM and a five-day PM + training (August–September 2021). Training of helpers (ToH) was hybrid and consisted of a two-day DWM and an eight-day PM + training (January-March 2022). Throughout the trial, helpers will receive weekly online supervision by these trainers. Trainers will receive online supervision by the Master trainers on an as-needed basis.

#### Treatment fidelity

Treatment fidelity will be assessed in various ways. First, helpers will fill in a session checklist after each DWM call and PM + session, and supervisors do so after each supervision. Second, 10% of the recordings of the DWM phone calls and PM + sessions will be assessed with fidelity checklists. This will be done for a random sample, stratified on helpers for DWM and PM + separately. Fidelity checks take place throughout the delivery of the programme, resulting in an iterative process of programme monitoring informing programme delivery. Third, metadata on participants’ app usage, e.g. if a participant has completed a module, will be collected.

### Care-as-usual (CAU)

CAU refers to all (mental) health care available to IMWs, both in and outside the Netherlands. IMWs become eligible for (mental) health care in the Netherlands upon signing up for health insurance, which is mandatory for all residents as well as those subject to payroll tax. They can access health care by registering with a general practitioner. No aspect of care will be altered or withheld as a result of participation in this study.

## Analysis

### Sample size

We aim to detect a small-to-medium Cohen’s *d* effect size of 0.3 on the PHQ-ADS composite score (primary outcome) at the *t*_*4*_ follow-up assessment point, which is based on previous RCTs on PM + [28, 29]. Power calculations suggested a minimum sample size of 74 per group (power = 0.95, α = 0.05, two-sided). With an expected 30% attrition at *t*_*4*_, we aim to include 212 study participants (intervention group: *n* = 106, control group: *n* = 106) over an 18-month inclusion period.

### Data analysis

Data will be analyzed once all data has been collected; no interim analyses will be conducted.

#### Quantitative data (RCT)

Intention-to-treat (ITT) and per-protocol (PP) analysis will be conducted for both the primary and secondary outcomes. ITT analysis will include all randomized participants (*N* = 212). PP analysis will include participants in the intervention group only if they have (a) clicked through at least three (out of five) modules of the DWM web application, and (b), if they qualified for PM + in terms of K10-distress score at *t*_2,_ attended at least four (out of five) PM + sessions. ITT analysis of the primary outcome will be used to answer the main research question.

To compare differences between the two treatment groups at baseline, t-tests (continuous variables) and chi-squared tests (categorical variables) will be conducted for normally distributed data. For continuous non-normally distributed data, Mann–Whitney tests will be conducted.

To estimate the treatment effect on the primary outcome at *t*_2_, *t*_3_, and *t*_4_, with *t*_4_ being our primary time point of interest, a linear mixed model will be used. This model will have time and treatment as fixed effects (as well as their interaction parameters), a baseline measure of the PHQ-ADS as a covariate, and subjects as random effects. The mean difference between the two treatment groups at each assessment with a 95% confidence interval will be obtained from this mixed model. The same linear mixed model will be used to estimate the effect of the DWM/PM + stepped-care programme on secondary outcomes: symptoms of depression (PHQ-9), anxiety (GAD-7), PTSD (PCL-5), quality of life (EQ-5D-5L), and an outcome-based resilience variable, operationalised as the PHQ-ADS total score against stressor exposure [78].

Missing data will not be imputed but will be treated as missing at random (MAR) as linear mixed models can handle missing data. If some items of a particular scale are missing (i.e. < 50%), the Corrected Item Mean Substitution method will be used [79]. This method uses the item mean across participants of the same study group and time point, weighted by the subject’s mean of completed items. All the above-mentioned analyses will be performed using Jamovi version 2.3.26 [80]. Jamovi is an open-source graphical user interface for R.

As an exploratory additional analysis, digital markers will be assessed using open-source tools in a Python environment, which is available on GitHub [38, 74]. Digital markers will be assessed using open source tools in a Python environment, which is available on GitHub [38, 74]. Change over time in digital markers will be calculated using repeated measures analysis of variance (ANOVA). A covariate mixed model of the primary endpoint will be conducted by adding relevant covariates at baseline, including gender, age, education, migration and work characteristics (e.g. duration of stay, type of contract), relationship status, living situation, life events, traumatic experiences, COVID-19-related events. An exploratory analysis will also be conducted to see if steroid hormone levels and baseline and between-session change in digital markers are associated with treatment recovery.

To determine the cost-effectiveness of the DWM/PM + stepped-care programme, health economic analysis will be conducted from a healthcare system and societal perspective. This will be conducted by focusing on the incremental cost per quality-adjusted life year (QALY, based on the EQ-5D-5L) and per change in PHQ-ADS composite score at the *t*_*4*_ follow-up assessment point (week 21). To do so, the total cost of the delivery of the intervention and changes in the uptake of health care will be assessed, using the CSRI. Between-group comparison of mean costs will be conducted using appropriate statistical tests, depending on the type and distribution of data. Univariate sensitivity analysis will be conducted using non-parametric bootstrapping to estimate the uncertainty and variability on trial parameters, which will be addressed by constructing cost-effectiveness planes and acceptability curves.

#### Qualitative data (cultural adaptation and process evaluation)

Interviews and FGD will be audio-recorded and transcribed verbatim. Transcripts will be analysed thematically through inductive coding using ATLAS.ti 23.1.2.

### Data management

All data is fully accessible to the VU research team. The management of data varies per study phase.

#### Quantitative data (RCT)

Data collected through Castor EDC is automatically pseudonymised. Data is fully accessible to the VU research team. Participants give separate permission for their data (a) to be shared with partners in the RESPOND project, and (b) to be stored at the research location for 15 years, so it can be used for future scientific research into the health of IMWs. Audio and video recordings of DWM support calls and PM + sessions respectively are stored pseudonymously in an active but secured data folder in Surfdrive (i.e., a cloud service for Dutch education and research) that can only be accessed by the members of the research team. At the end of the trial, when all hair samples are collected, they will be sent to the laboratory for analysis. Participants who submitted a hair sample will be informed of the analysis results of their sample. Participants can withdraw their consent to use their information at any time. Already collected data can still be used. Collected hair samples and recordings of DWM support calls and PM + video sessions that have not yet been analysed will be destroyed.

## Qualitative data (cultural adaptation and process evaluation)

Audio recordings of interviews and FGD will be destroyed after transcription. Pseudonymised transcripts will be stored for 10 or 15 years for respectively the cultural adaptation or the process evaluation. For the process evaluation, participants must separately give permission for the use of these stored transcripts in future scientific research into the health of migrant workers.

## Trial monitoring and adverse events reporting

The project has an Ethics and Data Advisory Board (EDAB), that will monitor and advise on data management, and ethical, legal, and societal issues that arise within the project.

(Serious) adverse events ((S)AEs) are defined as any undesirable experience occurring to a participant during the study, regardless of its connection to the study procedure or the DWM/PM + intervention. All SAEs will be recorded in Castor EDC and reported to the EDAB and the Medical Ethics Committee of the Amsterdam University Medical Center (UMC), location VU University Medical Center (VUmc). The research team will follow up with all SAEs until they are stabilized or have abated. If necessary, participants will be referred to a general practitioner.

Participants can withdraw from the study at any time. No withdrawal criteria have been stated. Based on reported (S)AEs, the principal investigator (PI) can decide to discontinue participation in the trial.

## Discussion

This article presents the study protocol for an RCT in which we aim to evaluate the (cost-) effectiveness of the remotely delivered, stepped-care DWM/PM + intervention among Polish migrant workers living in the Netherlands who have elevated levels of distress. This study is part of the larger EU H2020 RESPOND project, in which three other RCTs in Western Europe will also be conducted to test the effectiveness of this intervention amongst two other migrant populations (Italy [81], France as well as health care workers (Spain) [26].

As a result of the COVID-19 pandemic, the need for psychological interventions that target the most prevalent mental health problems has increased, particularly for vulnerable groups such as IMWs. In the Netherlands, IMWs experience multiple barriers to (mental) health care [82]. To our knowledge, this is the first RCT that combines two scalable, psychosocial WHO interventions into a stepped-care programme for IMWs. As it is offered as a stepped-care intervention, as IMWs may save time and money on mental health care this as the first step may be sufficient in improving mental health. By offering this stepped-care intervention in a remote format and in IMW’s native language, mental health care may become more accessible to a population facing practical challenges in seeing a mental health care professional in the country they are residing in. Additionally, as IMWs relocate regularly (both nationally and internationally), a remotely delivered intervention allows for continuous care. If proven to be effective, this offers the possibility for upscaling and implementing the DWM/PM + programme across European health care systems for IMWs and thereby bridging the treatment gap this population faces.

### Supplementary Information

**Additional file 1.**

**Additional file 2.**

## Data Availability

Data sharing does not apply to this article as no datasets were generated or analysed during the current study.

## Abbreviations

ACT: Acceptance and commitment therapy
AE: Adverse event
BTQ: Brief Trauma Questionnaire
CAU: Care-as-usual
CBT: Cognitive behaviour therapy
CSRI: Client Service Receipt Inventory
DHEA(S): Dehydroepiandrosterone (sulphate)
DIME: Design, Implementation, Monitoring and Evaluation
DWM: Doing What Matters in times of stress
eCAU: Enhanced care-as-usual (in this study PFA + CAU)
EDC: Electronic Data Capture
EQ-5D-5L: EuroQol 5-dimensional descriptive system—5-level version
GAD-7: Generalized Anxiety Disorder, seven-item measurement instrument
HCC: Hair cortisol concentrations
ICF: Informed consent form
IMW: International migrant worker
ITT: Intention-to-treat
K10: Kessler Psychological Distress Scale, 10-item measurement instrument
MAR: Missing at random
MIMIS: Mainz Inventory of Microstressors
PASS-content: Positive Appraisal Style Scale – content focused
PCL-5: PTSD Checklist for DSM-5, eight-item measurement instrument
PCL-C: PTSD Checklist for DSM-5, 20-item measurement instrument
PFA: Psychological First Aid
PHQ-9: Patient Health Questionnaire depression module, 9-item measurement instrument
PI: Principal investigator
PM +: Problem Management Plus
PP: Per-protocol
PTSD: Posttraumatic stress disorder
QALY: Quality-adjusted life year
RCT: Randomised controlled trial
SH +: Self Help Plus
ToH: Training of helpers
ToT: Training of trainers
UMC: University Medical Center
VUmc: VU University Medical Center
WHO: World Health Organization
