# Correction: Effectiveness of a scalable, remotely delivered stepped-care intervention to reduce symptoms of psychological distress among Polish migrant workers in the Netherlands: study protocol for the RESPOND randomised controlled trial

**DOI:** 10.1186/s12888-023-05475-4

**Published:** 2024-01-04

**Authors:** Rinske Roos, Anke B Witteveen, José Luis Ayuso-Mateos, Corrado Barbui, Richard A. Bryant, Mireia Felez-Nobrega, Natasha Figueiredo, Rafael Kalisch, Josep Maria Haro, David McDaid, Roberto Mediavilla, Maria Melchior, Pablo Nicaise, A-La Park, Papoula Petri-Romão, Marianna Purgato, Annemieke van Straten, Federico Tedeschi, James Underhill, Marit Sijbrandij, on behalf of the RESPOND Consortium

**Affiliations:** 1 Department of Clinical, Neuroand Developmental Psychology and WHO Collaborating Center for Research and Dissemination of Psychological Interventions, VU University, Amsterdam, Netherlands; 2 Department of Psychiatry, Universidad Autónoma de Madrid (UAM), Madrid, Spain; 3 Department of Psychiatry, La Princesa University Hospital, Instituto de Investigación Sanitaria Princesa (IIS-Princesa), Madrid, Spain; 4 Centro de Investigación Biomédica en Red de Salud Mental (CIBERSAM), Instituto de Salud Carlos III (ISCIII), Madrid, Spain; 5 Department of Neuroscience, Biomedicine and Movement Sciences, Section of Psychiatry, WHO Collaborating Centre for Research and Training in Mental Health and Service Evaluation, University of Verona, Verona, Italy; 6 School of Psychology, University of New South Wales, Sydney, NSW Australia; 7 Research and Development Unit, Parc Sanitari Sant Joan de Déu, Barcelona, Spain; 8 Equipe de Recherche en Epidémiologie Sociale (ERES), Institut Pierre Louis d’Epidémiologie et de Santé Publique (IPLESP), INSERM, Sorbonne Université Facult? de Médecine St Antoine, Paris, France; 9 Leibniz Institute for Resilience Research (LIR), Mainz, Germany; 10 Focus Program Translational Neuroscience (FTN), Neuroimaging Center (NIC), Johannes Gutenberg University Medical Center, Mainz, Germany; 11 Care Policy and Evaluation Centre, Department of Health Policy, London School of Economics and Political Science, London, UK; 12 Institute of Health and Society (IRSS), Université Catholique de Louvain, Brussels, Belgium; 13 Independent Research Consultant, Brighton, UK

**Correction: BMC Psychiatry (2023) 23:801**

10.1186/s12888-023-05288-5

Following the publication of the original article [1], the authors identified that the sentence under the subheading Sample size was incorrect. The correct sentence is given below.

The incorrect sentence is: Power calculations suggested a minimum sample size of 74 per group (power = 0.80, α = 0.05, two-sided).

The correct sentence is: Power calculations suggested a minimum sample size of 74 per group (power = 0.95, α = 0.05, two-sided).

The original article [1] has been corrected.
