# Supplementary material for: Effectiveness of a scalable, remotely delivered stepped-care intervention to reduce symptoms of psychological distress among Polish migrant workers in the Netherlands: study protocol for the RESPOND randomised controlled trial
Source: BMC Psychiatry. 2023 Nov 2;23:801. doi: 10.1186/s12888-023-05288-5 (PMC10623706; doi:10.1186/s12888-023-05288-5)
Supplement: Supplementary file 1 — Additional file 1. [file 12888_2023_5288_MOESM1_ESM.docx]

Information about the RESPOND research project

*English title:*

*Improving mental healthcare for labor migrants in the Netherlands during the COVID-19 pandemic: implementation of a stepped care program (DWM/PM+)*

Dear Sir/Madam,

With this information letter we would like to ask you if you would like to participate in medical-scientific research. We ask you to participate in a study on the feasibility and effectiveness of RESPOND, a step-by-step intervention for psychological symptoms of distress such as depression, stress and anxiety.

Participation is voluntary. To participate, your written permission is required.

Before you decide if you want to participate in this research, you will receive an explanation of what the research entails. In this letter, you can read what kind of research it is, what it means for you, and what the advantages and disadvantages are of participating in this research. It is a lot of information, but we ask you to please read this information carefully and then decide if you want to participate.

If you have any questions, please ask the researcher for an explanation. You can also ask the independent expert, who is mentioned at the end of this letter, for additional information. You can also talk about it with others, such as your partner, friends or family.

If you want to participate, you can fill in the form that you will find in Appendix B.

*Ask your questions*

You can make your decision based on the information you find in this information letter. In addition, we recommend that you do this:

- Ask questions to the researcher who gave you this information *[and who will call you next week]*.

- Talk to your partner, family or friends about this research.

- Ask questions to the independent expert, Ms Dominika Chromik

- Read the information on https://www.government.nl/topics/medical-research

# General information

The Vrije Universiteit (VU) Amsterdam has set up this research. Below we always call the VU Amsterdam the 'sponsor'. Investigators are conducting the study. This research requires 212 research participants. The medical ethics review committee VUmc has approved this research.

# Purpose and background of the study

In this study, we examine whether the step-by-step RESPOND intervention, which consists of the programs Doing What Matters (DWM) and Problem Management Plus (PM+) developed by the World Health Organization (WHO), reduces psychological symptoms of distress such as depression and anxiety.

# 3. What is the background of the investigation?

Depression, stress and anxiety are common psychological symptoms of distress among migrant workers. For example, these symptoms may have arisen due to limitations during the COVID-19 pandemic such as difficulty travelling back and forth to close family and friends in your home country, experiencing loneliness or deteriorating living or working conditions. Psychological distress can cause a lot of suffering and can lead to obstacles in carrying out daily activities.

Previous research shows that the programs we will investigate within RESPOND are effective in reducing mental health problems. With this research we want to find out whether the step-by-step provision of these programs is also a useful approach for migrant workers in the Netherlands.

# 4. How's the investigation going?

*How long does the investigation take?*

Are you participating in the investigation? Then that takes about 6 months in total.

*Step 1: Are you eligible to participate?*

First, we want to know if you are eligible to participate. That is why the researcher first asks you questions about symptoms of distress. We will set up a video call for this. This will take about 15 minutes. If it turns out that you are not eligible to participate, the researcher will tell you more about this.

*Step 2: the DWM program*

The DWM program is a program of a total of 5 weeks in which you go through a web application (app) via your smartphone or laptop/PC. When going through the chapters in the app, you will be given information about your symptoms of distress and you will be offered exercises to reduce them. You go through this app by yourself in your own time. In addition, you will be guided weekly via a remote helper by means of a short telephone contact (or if desired by means of in-app text messages). During this contact, the helper will ask you how the use of the app is going. If you have any questions about using the app, the helper will answer them.

*Step 3: The PM+ program*

The PM+ program is a program of a total of five weekly conversations by video call. The conversations are conducted by a trained helper who speaks your language (such as Polish). A PM+ conversation will take place in the language you master best and lasts 60 minutes. You will talk with the PM+ helper about symptoms of distress you may experience, such as anxiety and low mood. During the PM+ program you will receive information about these symptoms and you will do exercises to reduce stress reactions. For example, the PM+ helper will do breathing exercises with you. You will receive tips on how to deal with the symptoms of distress in daily life. In the PM+ conversations, techniques based on cognitive behavioral therapy are used. Cognitive behavioral therapy has been proven effective for reducing psychological symptoms of distress, such as depression and anxiety.

For this research we create 2 groups:

- **Group 1**. The people in this group get access to the 5-week DWM web application. One week after the DWM program ends, the people in this group will fill in the second questionnaire. Based on the answers to this questionnaire, the researchers will check if the symptoms of distress are still present. If distress is still present, these people are also offered 5 PM+ conversations with a helper. If the symptoms of distress have improved, they are not offered 5 PM+ conversations with a helper. In addition, they receive psychological first aid (PFA*) and can make use of the usual care.
- **Group 2**. The people in this group do not get access to the DWM web application or PM+ conversations. However, they do receive psychological first aid (PFA*) and can make use of the usual care.

* Psychological First Aid (PFA) is a one-off (telephone) contact developed by the WHO with a helper who offers a listening ear, support and practical tips.

A computer will decide which group you will enter. This is based on chance: you have 50% chance to enter Group 1 and 50% chance to enter Group 2. The researcher does not know whether you will be entered into Group 1 or Group 2. This is necessary to prevent this knowledge from influencing the results of the research. We would therefore ask you not to tell the researcher which group you are assigned to.

*Step 4: Examinations and measurements*

Questionnaires:
The study requires you to complete a number of online questionnaires 4 times over 6 months, with about 2 months between each questionnaire. Completing these questionnaires takes 45-60 minutes at a time. The questionnaires are about symptoms of distress, such as anxiety, low mood and stress. You will also receive questions about stressful situations and possible major events that you have experienced (in general and due to the COVID-19 pandemic), your general quality of life and use of healthcare.

Hair samples:

We also ask you for a sample of hair from your head. In hair we can measure hormones that are related to stress, such as cortisol, cortisone, and dehydroepiandrosterone (sulfate) (DHEA(S)). Often, people who experience more distress have different levels of these stress-related hormones. We can analyze this in the hair. Hair grows about 1 cm per month; a hair sample of, for example, 3 cm informs us about the amount of stress-related hormones during these 3 months.

In RESPOND, we want to examine if the DWM/PM+ program, which is designed to reduce distress, also has an effect on these stress-related hormones. We also want to examine if there is a difference in these stress-hormones between the people who are and who are not eligible to participate. Therefore, if you are not eligible to participate in the study, we still ask you for a hair sample:

- We ask people who are eligible to participate for two hair samples: one at the start of the study, and one at the end.
- We ask people who are not eligible to participate in the study for one hair sample only. If you are not eligible to participate, but still give a hair sample, we also ask you to fill in one questionnaire.

Every time you give a hair sample, we also ask you to answers questions about your hair (e.g. how often you wash your hair?) and questions about substance use which influence these hormones (e.g. if you have taken corticosteroids).

If you give a hair sample, we will inform you about the result of your hair sample. Giving a piece of the hair of your head is entirely voluntary. You can also participate in the RESPOND study without agreeing to give a sample of your hair. In the consent form you can indicate if you give us permission for this.

Digital markers:

We ask your permission to video record the PM+ conversations, which will be through video call. We want to use these video records to analyze your facial activity, voice and movement, which are called digital markers. Such digital markers have been linked to psychological functioning. Nowadays, if we want to know how someone is doing psychologically, people need to fill in questionnaires. Filling in questionnaires and analyzing them takes a lot of time. If we can use digital markers instead of questionnaires, this could save a lot of time.

There is also a lot of potential to use digital markers: our lives are becoming more digital as people video call with family, friends, and since the start of the COVID-19 also more with mental health care workers. However, before we can use digital markers to assess how someone is doing psychologically, more research needs to be conducted. In RESPOND, we want to examine if the DWM/PM+ program, which is designed to reduce distress, also has an effect on digital markers such as facial activity, voice, and movement.

Video recording the PM+ conversations is entirely voluntary. You can also participate in the RESPOND study without agreeing to video record the PM+ conversations. In the consent form you can indicate if you give us permission for this.

# 5. What agreements do we make with you?

We'd like the investigation to go well. That is why we make the following agreements with you:

- You take part in every (telephone or video call) appointment.
- You fill in all (online) questionnaires.
- You contact the researcher in these situations:
  - You will be admitted to or treated in a hospital.
  - You suddenly have problems with your health.
  - You don't want to be part of the investigation anymore.
  - Your phone number, address or e-mail address changes.

# 6. What are the advantages and disadvantages when you participate in the research?

Participating in the study can have advantages and disadvantages. We list them below. Think about this carefully and you can also talk about it with others.

The step-by-step RESPOND intervention, consisting of DWM and PM+, may reduce symptoms of distress, such as anxiety, low mood and stress, but this is not certain. If you enter the control group (Group 2), you yourself will not benefit from participating in this study. However, with your participation you help the researchers to gain more insight into whether short-term mental health programs reduce symptoms of distress and improve wellbeing among migrant workers in the Netherlands. If you give permission to video record the PM+ conversations, you also help the researchers gain more insight into digital markers.

Taking part in the study can have disadvantages:

- Participating in the study costs you (extra) time.
- Completing questionnaires can cause temporary tension or be confrontational. You can always skip questions if you prefer.
- You may temporarily experience more tension or anxiety while going through the programs. The helpers for the programs in RESPOND are trained to deal with feelings of tension and anxiety. There is also always an experienced employee available who can provide psychosocial support or can refer you to more specialized care if you wish.
- You must adhere to the agreements that are part of the research.
- If you give a hair sample, you lose some hairs. Each hair sample is about as thick as half a pencil.

*Do you not want to participate?*

You decide if you participate in the study. Do you not want to participate? The researcher can tell you more about treatment options available, such as psychological support, and their advantages and disadvantages.

# 7. When will the research end?

There might be situations in which the researcher will let you know that research ends for you.

In these situations, the research will end for you:

- When we ask questions to check if you are eligible to participate (as explained under section 4, Step 1), you indicate that you do not experience much psychological distress, such as depression or anxiety, or daily limitations;
- If you are not eligible but still want to give a hair sample, the research ends after you gave the hair sample and filled in the online questionnaire.
- The 4 appointments for completing the online questionnaires are completed;
- You want to stop the research yourself. That is allowed at any time. Report this immediately to the researcher. You do not have to tell us why you are stopping;
- The investigator thinks it is better for you to stop. The researcher will still invite you for a meeting to discuss this;
- One of the following authorities decides to stop the investigation:
  - the sponsor
  - the government, or
  - the medical ethics committee that assesses the research.

*What happens if the research ends for you?*

The researchers use the data that has been collected up to the moment of stopping.

The entire study is over when all participants have completed the study.

# 8. What happens after the research?

About 2 years after your participation, the researcher will inform you by e-mail about the main results of the study.

If you need more psychological help after this intervention has ended for you, we will refer you to your general practitioner. In case you do not have a general practitioner, the research team will make sure that you get an appointment with one.

# 9. What do we do with your data?

Are you participating in the study? Then you also give permission to collect, use and store your information.

*What information do we store?*

We store this information:

- your name

- your gender

- your address

- your telephone number

- information about your health

- user data of the application

- audio records of the DWM phone calls (if you give us permission)

- video record of the PM+ conversations (if you give us permission)

- hair samples of the first and fourth appointment for filling in the questionnaires (if you give us permission)

*Why do we collect, use and store your information?*

We collect, use and store your information to answer the questions of this research and to publish the results. The helper can also see your progress in the DWM app and answers you write to questions in the app. This way, the helper can support you better during the weekly phone call. Furthermore, we want to make an audio record of the DWM phone calls. This way we can check if the helper performs the DWM phone calls the way that they are supposed to (fidelity checks). If you give us permission to video record the PM+ conversations to analyze them for digital markers, we can also check if the helper performs the PM+ conversations the wat they are supposed to (fidelity checks). In the consent form you can state if you give us permission to make these audio and video records for DWM and PM+. This is not obligated. By doing this, we take your privacy into account (see below).

*How do we protect your privacy?*

To protect your privacy, we give your information, including audio/video records and/or hair sample(s), a code. We keep the key to the code in a secure place at the research institution. When we process your data, we always only use that code. Also, in reports and publications about the research no one can recall that it was about you. The coded hair samples will be sent to a laboratory in Germany (Dresden LabService GmbH).

*Who can see your information?*

RESPOND project:

Some people can view your name and other personal information without a code. These are people who check whether the researchers are carrying out the research properly and reliably. These persons can access your information:

- Members of the committee that monitors the safety of the investigation.
- An auditor hired by the sponsor or a controller who works for the Amsterdam Public Health research institute (APH).
- National and international supervisory authorities. For example, the Health Care Inspectorate (Inspectie Gezondheidszorg en Jeugd, IGJ).

These persons keep your information secret. We ask you to give permission for this access.

DWM program (app):

When processing your personal data, we use Ipportunities BV, Arnhem as service provider (processors) who processes your personal data on behalf of and under the responsibility of the Stichting VU. The Stichting VU concludes a processing agreement with this service provider to ensure that your personal data is processed carefully, securely and in accordance with the General Data Protection Regulation (GDPR). We remain solely responsible for these processing activities.

The DWM mobile website does not use Google Analytics. Although a Youtube video is embedded in the first module, this is done through the privacy enhanced method that Youtube offers, which leaves no cookies (i.e. https://www.youtube-nocookie.com instead of <https://www.youtube.com>).

*How long do we store your information?*

We store your data for 15 years at the location of the sponsor. The audio records of the DWM phone calls will be stored until the end of the research and we have performed all fidelity checks. After this, audio records will be destroyed. If you give us permission to make video records of the PM+ conversations, then they are stored for 15 years at the location of the sponsor. Hair samples are destroyed immediately after we have analyzed them.

*We use your data for other research within the European Union*

In this research, we also store your coded data in order to merge it with research data from researchers from other countries in the European Union (EU) and the United Kingdom (UK).

Your privacy is protected to an appropriate level in these countries. The data must be shared encrypted and without your name and other personal information that can directly identify you.

*Can we use your information for other research?*

At the end of this research, we want to do follow-up research with participants, including those whose participation has already ended. This will consist of interviews with participants in this study and their family members/close friends about their experience with this program. In the consent form you indicate whether you agree with this. Do you not give permission? Then you can still participate in this study.

In addition to follow-up research, your data may also be important for other scientific research into the health of migrant workers. For this reason, your data will be stored in the research institution for 15 years. In the consent form you indicate whether you agree with this. Do you not give permission? Then you can still participate in this study.

*Can you withdraw your consent to the use of your information?*

You can withdraw your consent to the use of your information at any time. This applies to the use in this research and to use in other research. But beware: are you withdrawing your consent, and have researchers already collected data? Then they may still use this information.

We will analyze the hair samples and video recorded PM+ conversations once the study is finished and we have collected all hair samples and video records. The researchers will destroy the collected hair samples and/or video records after you withdraw your permission. But are your hair samples and/or video records already analyzed and have we collected data? Then the researcher may continue to use its results.

*Would you like to know more about your privacy?*

- Would you like to know more about your rights regarding the processing of personal information? Please go to: [www.autoriteitpersoonsgegevens.nl](http://www.autoriteitpersoonsgegevens.nl).
- Do you have questions about your rights? Or do you have a concern about the processing of your personal information? Please contact the person responsible for the processing of your personal information. For this research, this is VU Amsterdam. See Appendix A for contact details and website.
- If you have any concerns about the processing of your personal information, we recommend that you first discuss them with the research team. You can also contact the Data Protection Officer of VU Amsterdam. Or you can submit a complaint to the Dutch Data Protection Authority.

*Where can you find more information about the research?*

You will find more information about the research on the following website: http://www.trialregister.nl/trialreg/. After the research, the website may show a summary of the results of this research. You can find the research by searching for "NL9630".

# 10. Do you receive compensation if you participate in the study?

Participation in the RESPOND study is free of charge. To participate in this study, you will receive a voucher of € 10.00 for completing each of the four (online) questionnaires. In addition, you will be reimbursed for travel expenses in case you come to VU University for research measurements. If you are not eligible to participate in the RESPOND study, but you still give a hair sample and fill in one (online) questionnaire, then you receive one voucher of € 10.00 for completing the questionnaire. You do not receive more compensation if you give hair samples or give permission to record the PM+ conversations. Will you stop before the research is completed? Then you will receive a compensation only for the questionnaires you completed.

# 11. Are you insured during the study?

You are not additionally insured for this study. Because taking part in the study has no additional risks. That is why the sponsor of the METc VUmc does not have to take out additional insurance.

# 12. Do you have any questions?

You can ask questions about the research to the VU research team. Would you like advice from someone who has no benefit in it? Then go to Ms. Dominika Chromik. He knows a lot about the research, but does not participate in this research. Do you have a complaint? Then discuss this with the researcher. Would you rather not do so? Then go to the complaints officer (Prof. Dr. Annemieke van Straten). Appendix A tells you where to find them.

# 13. How do you give permission for the research?

First, you can think carefully about this research (at least one week). After that, you tell the researcher if you understand the information and if you want to participate or not. Would you like to participate? Then you fill in the consent form that is attached to this information letter. Both you and the researcher will receive a signed version of this consent form.

Thank you for your time.

# 14. Appendices to this information letter

A. Contact information

B. Consent form

# Appendix A: Contact information

## Onderzoekers VU Amsterdam

## Executive researcher:

Ms. Rinske Roos, r.roos@vu.nl 020-598 58 48

Available: Monday to Friday between 9.00 a.m. and 5.00 p.m.

Main researchers:

Ms. Dr. Anke Witteveen, a.b.witteveen@vu.nl

Ms. Prof. Dr. Marit Sijbrandij, e.m.sijbrandij@vu.nl

**Independent expert:** Ms. Dominika Chromik, d.chromik@ggzkeizersgracht.nl

**Complaints officer**: Ms. Prof. Dr. Annemieke van Straten, a.van.straten@vu.nl

**Data protection officer**: Data protection officer VU, functionarisgegevensbescherming@vu.nl

# Appendix B: Consent form

*RESPOND: Improving mental health care for migrant workers in the Netherlands
during the COVID-19 pandemic.*

- I have read the information letter. I could also ask questions. My questions have been sufficiently answered. I had enough time to decide whether to participate.
- I know that participation is voluntary. I also know that I can decide at any time not to participate or to discontinue the study. I do not have to give a reason for that.
- I know that I can be referred to a general practitioner if I need additional psychological support.
- I know that some people can view my information. Those people are listed in this information letter.
- I give permission for the collection and use of my information in the way and for the purposes stated in the information letter (see also section 4).
- I give permission to keep my data at the research location for 15 years after this.
- I give permission for the sharing of my information in the context of this study with partners in the RESPOND project in countries within the EU and the UK. I have been informed that my privacy is protected to an appropriate level in these countries. The data must be shared encrypted and without my name and other personal information that can directly identify me.
- I **□ do give**

**□ do not give**

permission to make an audio record of the support phone calls with a helper during the DWM program (app) and store them until the end of this study, so that they can be used for fidelity checks.

- I **□ do give**

**□ do not give**

permission to make a video record of the PM+ conversations and store them for 15 years, so they can be used for digital analysis of facial, vocal, and movement behavior.and for fidelity checks.

- I **□ do give**

**□ do not give**

permission to collect hair samples in order to answer the research question of this study. I am aware that my coded hair sample is sent to Germany for analysis, after which they will be destroyed. Stress-related hormone levels will be stored for 15 years.

- I **□ do give**

**□ do not give**

permission to approach me again after this research for a follow-up research.

- I **□ do give**

**□ do not give**

permission to keep my data at the research location for 15 years after this, so that it can be used for future scientific research into the health of migrant workers.

- I want to participate in this research.

Name participant: _________________________

Signature participant: _________________________

Date: ___ / ___ / ______ (DD/MM/YYYY)

I declare that I have fully informed this participant about the mentioned research.

If information becomes known during the study that could influence the participant’s consent, I will inform him / her in good time.

Name researcher (or its representative): _________________________

Signature researcher (or its representative): _________________________

Date: ___ / ___ / ______ (DD/MM/YYYY)

*The participant will receive a complete information letter, together with a copy of the signed consent form.*
